# Assessing behavioral reallocation after acute environmental manipulations using an asymmetric cocaine versus sucrose choice task in male and female rats

**DOI:** 10.21203/rs.3.rs-7802392/v1

**Published:** 2025-10-30

**Authors:** David B. Nowak, Mary K. Estes, Bailey E. Schultz, Robert A. Wheeler, John R. Mantsch

**Affiliations:** 1 Department of Pharmacology and Toxicology, Medical College of Wisconsin, Milwaukee, WI USA;; 2 Medical Scientist Training Program, Medical College of Wisconsin, Milwaukee, WI USA;; 3 Department of Biomedical Sciences, Marquette University, Milwaukee, WI, USA

**Keywords:** cocaine choice, cocaine self-administration, sucrose self-administration, behavioral pharmacology

## Abstract

**Rationale:**

Maladaptive drug choice is a defining component of substance use disorders; whereby drug misuse persists despite adverse consequences. A goal of behavioral interventions, such as contingency management programs, is to promote reallocation of behavior towards adaptive pursuits. The neurobiological mechanisms that govern behavioral reallocation in the context of ongoing cocaine use remain poorly understood.

**Objective:**

Here, we demonstrate a cocaine versus sucrose choice paradigm in male and female rats using a novel format that dissuades exclusive sucrose or cocaine choice by offering sucrose pellets on a progressive ratio, while cocaine infusions are offered at a low, fixed ratio. Maintenance of both drug and non-drug choice will allow for the investigation of behavioral reallocation following environmental or pharmacological manipulations.

**Materials and methods:**

In experiment 1, rats were trained to perform the choice task and were tested under conditions of acute food restriction followed by cocaine non-reward (extinction). In experiment 2, rats were trained to perform the choice task and then subject to a cocaine punishment contingency, whereby cocaine choice was punished by an aversive white noise (AWN).

**Results:**

Rats display cocaine dose- and sucrose price-dependent choice preference with no apparent sex differences. However, after either 24 hours of food restriction or removal of the cocaine reinforcer, male rats reallocate behavior towards sucrose, while female rats do not. Likewise, AWN-punished cocaine choice drives reallocation towards sucrose in male rats, but not females.

**Conclusion:**

Our data suggest that male and female rats exhibit unique reallocation strategies in response to changing contingencies.

## Introduction:

Choice is a ubiquitous feature of everyday life, and for individuals aiming to reduce or stop their drug use, decisions regarding drug use occur in a dynamic marketplace, with options defined by their expected value and price (i.e., effort). However, a common symptom of substance use disorders is the disrupted ability to assign proper value to drug reinforcers relative to available alternatives (Goldstein and Volkow 2002). Heightened salience attributed to highly reinforcing drugs makes drug choice easy to underwrite, despite the serious risks to personal and societal wellbeing. The neurobiological mechanisms that drive drug use while simultaneously diminishing motivation for alternative reinforcers remain poorly understood.

Preclinical models have sought to understand the neurobiology of drug seeking and taking behavior that represent pathological patterns in humans. Nevertheless, most of what has been described about addiction-like behavior has been gleaned from “drug-only” self-administration studies, which measure motivation to obtain drug in the absence of an alternative reinforcer. Since the decision to use or seek drug in a traditional “drug only” self-administration paradigm provide little to no opportunity cost (i.e., something to lose), heightened motivation for drug, or attenuation thereof, need not describe pathology, but rather an increase or decrease in general goal directed behavior. Preclinical drug choice models have emerged as valuable tools for disentangling motivation for commonly misused drugs in the presence of alternative reinforcers (Lenoir, Serre, Cantin, and Ahmed 2007; Kerstetter et al. 2012; Townsend et al. 2021; Thomsen et al. 2008; Thomsen, Barrett, Negus, and Caine 2013; Thomsen, Fulton, and Caine 2014; Thomsen et al. 2017; Marcus, Negus, and Banks 2022; Marcus and Banks 2023; Marcus et al. 2024; Sedighim et al. 2021; Venniro, Panlilio, Epstein, and Shaham 2021; Beckmann, Chow, and Hutsell 2019; Chow, Hofford, and Beckmann 2021; Beasley et al. 2024; Cantin et al. 2010). Choice models have allowed researchers to observe how rodents and non-human primates allocate behavior depending on unique economic contexts, which may provide clues as to what genetic or environmental factors facilitate heightened motivation for drug use.

Although there are no FDA approved medications for treatment of stimulant use disorders (StUDs), behavioral interventions such as contingency management programs have shown marked benefit (Coughlin et al. 2025). Contingency management programs offer tangible reinforcers, often small monetary prizes, in exchange for biochemically verified drug abstinence (DePhilippis et al. 2018). Preclinical choice studies bear resemblance to contingency management interventions by offering alternative reinforcers in lieu of drug access. In recent years, several reports have indicated that rats tend to prefer non-drug reinforcers to drug reinforcers often resulting in a state of voluntary drug abstinence (Venniro et al. 2018; Venniro, Panlilio, Epstein, and Shaham 2021; Lenoir, Serre, Cantin, and Ahmed 2007; Caprioli et al. 2015). The finding that rodents readily relinquish the opportunity to self-administer commonly misused stimulants, alcohol and opioids, in favor of alternative reinforcers like social interaction or palatable food, has raised questions about the construct validity of rodent models of addiction since relapse tends to persist in human populations. Nevertheless, others have reported that rodents will reverse their reinforcer preference in favor of drugs, including cocaine, when either the dose of drug or the effort requirement for the non-drug reinforcer is sufficiently increased (Cantin et al. 2010; Thomsen, Barrett, Negus, and Caine 2013). By manipulating the economic context of drug choice, drug choice models offer a valuable tool for studying the relative motivation for drug versus non-drug reinforcers.

Still, drug choice models often require complex training and testing schemes, which hinders their use for investigators who may be primarily interested in the neurobiological correlates of drug choice. Further development of choice models is needed to create schemes that are relatively simple to implement, allow for observation of mixed choice, and produce interpretable outcomes. Additionally, few rodent studies have investigated, and less have reported, sex differences in drug choice behavior (Kerstetter et al. 2012; Stocco et al. 2025). Notably, evidence suggests that females, as a consequence of gonadal estrogen signaling, are more likely to choose cocaine versus food than males (Kerstetter et al. 2012). Similarly, estrogen supplementation in males is sufficient to increase cocaine choice (Bagley et al. 2019). And while it may be true that male and female rats generally express little difference in choice behavior when both options are predictable, there is evidence to suggest that males and females may allocate behavior differently when the choice context is altered or punishment is encountered. For example, increasing the inter-trial interval uncovered a sex difference whereby female rats increased methamphetamine choice over food, when no sex difference was apparent using a shorter inter-trial interval (Stocco et al. 2025). Risky decision-making tasks may also provide insight into the idea of emergent sex differences. When allowed to choose between a small reward or a large reward contingent upon a probabilistic punishment, female rats tend to both avoid the large, risky choice and omit more trials than males (Orsini et al. 2016). Moreover, when foraging behavior is punished by a probabilistic foot shock, male rats cope by increasing the size of their feedings, while females reduce the amount of time foraging at the expense of physiologic needs (Chowdhury et al. 2019). Together, these studies indicate that sex differences may emerge after acute contingency changes and that female rats may struggle to maximize utility when attempting to mitigate risk.

Building upon previous work, we devised a discrete trial-based choice procedure that employs an asymmetrical reinforcer schedule, such that sucrose pellets are offered on a progressive ratio, while cocaine infusions are offered steadily on a low, fixed-ratio. This format encourages early sucrose choice and late cocaine choice, allowing rats to respond under a continuum of relative costs during a single session. Since we were able to elicit a mixture of both sucrose and cocaine choice behavior within a single session, we could assess how environmental manipulations affect reinforcer preference. Following the establishment of baseline choice behavior, we initiated several environmental manipulations including acute food restriction, omission of the cocaine reinforcer (non-reward), and aversive white noise-punished cocaine choice. To our knowledge, there are no reports of white noise-punished cocaine choice, offering novel insight into behavioral reallocation under conditions of punished drug choice. The present study seeks to characterize the effects of cocaine dose, feeding status, cocaine non-reward, biological sex, and punishment contingencies on behavioral allocation in the context of asymmetric reinforcer requirements.

## Materials and Methods:

### Animal subjects

Adult male and female Sprague Dawley rats were obtained from Envigo (Indianapolis, IN, USA) and individually housed upon arrival. Additionally, four female Long Evans rats bred in a colony at the Medical College of Wisconsin were used for the experiment investigating effects of satiety on choice behavior. The vivarium was regulated on a reversed light/dark cycle (12hr/12hr). Average female weight upon study initiation was 259.7 (SEM +/− 5.20) g and the average male weight was 385.5 (SEM +/− 5.81) g. Animals in Experiment 1 were allowed *ad libitum* access to standard rat chow (Inotiv Teklad Diet) and water, except for the 24 hours preceding the food-restricted choice session. Animals in Experiment 2 were food restricted to achieve body weight stability (~95% of starting weight) during the initial food and cocaine training sessions but returned to *ad libitum* access during fixed-ratio 3 (FR3) cocaine sessions and all subsequent choice sessions. Experiments were carried out during the dark phase and approved by the Institutional Animal Care and Use Committee (IACUC) at the Medical College of Wisconsin (MCW) in accordance with NIH Guide for the Care and Use of Laboratory Animals.

### Drugs

Cocaine hydrochloride was acquired through the NIDA Drug Supply Program. Cocaine salt was diluted in bacteriostatic heparinized saline and delivered intravenously at a concentration of 1 mg/mL. Dosing was determined by infusion duration delivered by a computer controlled single speed pump (Med Associates; St. Albans, VT). Methohexital (0.1mL, i.v., Brevital; Henry Schein Inc.) was used to assess catheter patency for each animal at the terminus of each experiment.

### Experiment 1: Cocaine dose effects on choice allocation in male and female rats

#### Initial PR sucrose training

Rats were placed into operant conditioning chambers (Med Associates, St. Albans, VT) for 2 hours each day to acquire lever pressing behavior reinforced by 45-mg unflavored sucrose pellets (BioServ). During the initial time-based training phase, a single sucrose pellet was earned under a progressive ratio (PR) schedule of reinforcement with the following sequential response requirements: 1, 2, 3, 4, 5, 6, 7, 8, 9, 10, 12, 14, 16, 18, 20, 25, …, 95, 100 (max 31 pellets per session). We chose this custom linear schedule versus more commonly used exponential requirements due to the time constraints of a discrete trial procedure and a desire to bias early sucrose choice. A solid cue light was illuminated above the sucrose lever to signal the availability of the sucrose pellet reinforcer. Upon completion of a ratio requirement, the lever was retracted, the cue light was extinguished, and a single pellet was dispensed into a food receptacle adjacent to the lever. A timeout of 10 seconds occurred after the pellet was delivered during which an indirect “house light” was illuminated. The requirement to advance to the next training step required rats to respond on the sucrose lever more than 100 times/session for two consecutive sessions.

#### Discrete trial sucrose-only training

**Figure 1.**
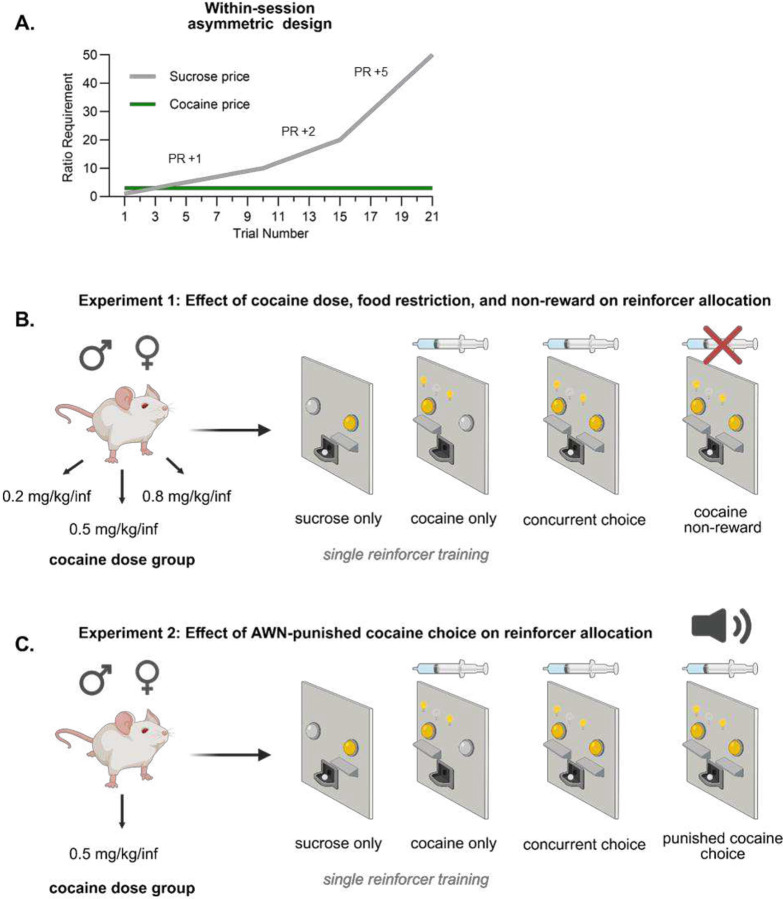
Description of asymmetric reinforcer schedule and experimental overview Each choice session consisted of 21 discrete trials during which the effort requirement for sucrose increased after each trial and the effort requirement for cocaine remained constant at a fixed ratio of 3 (A). Experiment 1 involved three groups of male and female rats, each receiving a unique cocaine dose (0.2, 0.5, and 0.8 mg/kg/infusion, respectively). After testing choice behavior at baseline conditions rats were tested for choice behavior after acute food restriction and a cocaine non-reward contingency (B). A separate cohort of male and female rats were used for Experiment 2. These rats were exposed only to the 0.5 mg/kg/infusion dose of cocaine and underwent a slightly abbreviated training protocol. After testing under baseline choice conditions, these rats were subject to a punished cocaine choice contingency, where cocaine choice was accompanied by a 10s burst of 90dB white noise (C).

To familiarize rats with the discrete trial procedure, they underwent four sucrose-only discrete trial sessions. These sessions mirror the later described “choice sessions” except that only the sucrose lever was inserted and the solid cue light above the sucrose lever was illuminated. The side assigned to the sucrose lever was counterbalanced among animals throughout the experiment (see Fig. 1A for lever locations). Each session consisted of 21 discrete trials. Each trial began with the insertion of the sucrose lever and illumination of the sucrose cue light. Rats were allowed up to 7 minutes to fulfill the ratio requirement for a single sucrose pellet before the trial terminated. The ratio requirement for each trial was: 1, 2, 3, 4, 5, 6, 7, 8, 9, 10, 12, 14, 16, 18, 20, 25, 30, 35, 40, 45, 50. After the ratio requirement was completed or 7 minutes had elapsed (whichever occurred first), the lever retracted and cue light extinguished, while an indirect “house light” illuminated and a 2-minute timeout period began. Any trials forgone due to time expiration were recorded as omissions. A pellet was not dispensed if the result of the trial was an omission. *Regardless of success on the previous trial*, the response requirement for a sucrose pellet increased when each new trial began. Animals completed two sucrose-only discrete trial sessions before undergoing intravenous catheter implantation. After a 5-day post-operative period, rats completed the remaining two sucrose-only discrete trial sessions before beginning cocaine-only training. Sucrose pellet dispensers were tested manually by the experimenter before and after each session. Although rare, if the dispenser was found to be stuck/inoperative at the end of a session, data from that session were excluded from analysis.

#### Initial cocaine self-administration training and maintenance

For Experiment 1, three different cocaine doses were administered to three different groups of male and female rats. The doses were low (0.2 mg/kg/inf), medium (0.5 mg/kg/inf), and high (0.8 mg/kg/inf). Each rat experienced only one dose of cocaine for the duration of the experiment. Rats from the 0.5 mg/kg/inf dose group underwent further testing to examine environmental manipulations including food restriction and cocaine non-reward.

Following the last sucrose-only discrete trial session, rats were returned to the same operant chambers for cocaine-only training. The lever flanking the opposite side of the food hopper relative to the sucrose lever was inserted at the beginning of each session crowned by a flashing cue light (0.5s on, 0.5s off) to serve as a discriminative stimulus for cocaine availability. Following a single lever press on the cocaine lever, an infusion was delivered accompanied by illumination of the house light, retraction of the cocaine lever, and a timeout period of 20s. After 2 consecutive days of >10 infusions earned on the FR1 schedule, rats proceeded to FR3 cocaine training for an additional 13 days. Each training session lasted 2 hours or until 50 infusions were achieved. The cocaine-only maintenance period consisted of 13 daily sessions, however after sessions 7 and 10, a “probe” choice session was offered.

#### Discrete trial choice sessions: Probe and continuous choice sessions

To familiarize rats with the concurrent reinforcer format, two “probe” choice sessions were offered during the “cocaine-only” training phase. The probe choice sessions were identical to the continuous choice sessions offered following the last day of cocaine-only training during the continuous choice period. The 21-trial “choice session” format was identical to the “discrete trial sucrose-only training” sessions described above except that both reinforcer levers and accompanying cue lights were presented at the onset of each trial. Sucrose pellets were earned under a progressive ratio (defined above), while cocaine infusions were always offered under a FR3 schedule. Each trial consisted of a 7-minute response window during which rats could respond on either lever. Responses on each lever were non-mutually exclusive (i.e., the sucrose lever did not “lock out” or reset after a response on the cocaine lever and vice versa). Following reinforcer selection or omission (due to response window expiration), a 2-minute timeout period ensued, marked by lever retraction and house light illumination. The next trial was signaled by insertion of both reinforcer-associated levers and their respective cue lights. Importantly, the response requirement for sucrose increased at the start of each trial *independently* of the previous trial result, while the cocaine requirement remained at FR3. Following the last day of cocaine-only training, rats underwent nine daily choice sessions (continuous choice session phase). Reinforcer selection or omission was recorded for each trial.

#### Choice session manipulations

Two separate manipulations were administered following the 9-session choice period for the rats in the medium dose group (0.5 mg/kg/inf). The first manipulation involved acute home cage food restriction for 24 hours prior to an additional choice session. The rationale behind this intervention was that a state of food restriction may inflate the relative value of sucrose pellets to cocaine infusions. Following the nine-session continuous choice period, home cage food was withheld for 24 hours prior to the 10^th^ choice session. The “food restriction test” occurred in same manner as the other choice sessions. Ad libitum food was restored immediately after this choice session. Importantly, on an “off day” immediately following the food restriction session, all animals were tested for catheter patency via intravenous methohexital administration to ensure that cocaine infusions could be properly delivered. To examine the effects of satiety on choice, we trained a small group of Long Evans female rats (n=4) using our choice protocol and provided them with ten minutes of unlimited access to sucrose pellets in the operant conditioning chamber prior to testing for cocaine (0.5 mg/kg/infusion) vs. sucrose choice (Fig. S3).

The second manipulation involved withholding cocaine infusions to create cocaine non-reward conditions. The rationale behind this manipulation was to investigate whether rats could update their choice allocation when confronted with non-reward or if choice patterns would persist (i.e., habitual responding). A subset of animals underwent this manipulation during an additional two choice sessions under cocaine non-reward conditions to assess the effect of cocaine omission on choice behavior. These sessions were identical to the continuous choice sessions except that cocaine was not delivered upon completion of the ratio requirement for the cocaine reinforcer.

### Experiment 2: Effects of aversive white noise-punished cocaine choice on reinforcer allocation in male and female rats

#### Abbreviated sucrose and cocaine training

A separate cohort of male and female rats were used to assess the effects of aversive white noise (AWN)-coupled cocaine infusions on choice behavior. Rats were trained in a similar manner to those in Exp. 1, with a few exceptions. The first difference is that rats were food restricted daily to maintain ~95% of their starting body weight throughout the single reinforcer training periods to facilitate operant learning. Animals were allowed *ad libitum* food access prior to catheter surgery and during the post-operative period. The second difference is that rats were trained to respond for sucrose pellets under a FR1 schedule, initially. Once rats achieved at least 50 responses in a single 2-hour FR1 session, they were promoted to time-based and subsequent discrete-trial PR sucrose training, which proceeded as described in Exp. 1. Both adjustments, mild food restriction and initial FR sucrose training, were intended to shorten the duration of operant training. Cocaine-only training was identical to Exp. 1 except that, following the FR1 acquisition period, rats underwent only 7 sessions of FR3 cocaine-only SA (versus 13 sessions in Exp. 1; ad libitum food available during 7 FR3 cocaine-only sessions). All animals in Exp. 2 received cocaine at the 0.5 mg/kg/inf dose.

#### Concurrent choice sessions and manipulations: Aversive white noise (AWN) and cocaine extinction

Following single-reinforcer training, rats underwent seven concurrent choice sessions. These choice sessions were identical to those described above in Exp. 1. On the day following the 7^th^ choice session, a choice session was administered during which each cocaine choice was paired with the activation of aversive white noise (10s, 90dB; Noise generator, Med Associates, St. Albans, VT). Our lab and others have used this versatile aversive stimulus that is easy to implement and supports punishment, negative reinforcement, and physiological signs of aversion (Goedhoop et al. 2022; Grafelman et al. 2025). This punishment was intended to reduce cocaine choice and encourage reallocation towards unpunished sucrose choice. On the day following introduction to this new contingency, rats underwent a second session with AWN-punished cocaine choice to assess behavior after having learned the new outcome. Catheter patency was confirmed prior to study conclusion via intravenous methohexital infusion and subsequent loss of righting reflex.

### Statistical considerations

Data from each choice session were first examined for normality using the Shapiro-Wilk test. Widespread violations of the assumption of normality precluded parametric analyses. Therefore, we implemented non-parametric testing scheme for most dependent measures including # of cocaine infusions, # of sucrose pellets, # of omitted trials, terminal sucrose trial, and latency to cocaine choice. For food restriction, non-reward, and punished cocaine experiments, we calculated a difference score (value after manipulation – value before manipulation) for each dependent measure. The distribution of difference scores satisfied normality assumptions and therefore parametric analyses were used. Statistical tests were conducted using GraphPad Prism 10 (Boston, MA, USA).

## Results:

### Effects of progressive sucrose price and cocaine dose on cocaine vs. sucrose choice

All choice data were evaluated to determine if assumptions for parametric testing were met. Due to the fundamentally bounded nature of the discrete choice trial limit, results of the Shapiro-Wilk test revealed that data frequently did not follow a Gaussian distribution. Thus, we were constrained to non-parametric comparisons between doses and sexes, separately.

Following single-reinforcer training and introduction to the choice session format via probe sessions, rats were exposed to daily choice sessions to determine the impact of different cocaine doses on reinforcer preference (Fig. S1A). Each group of rats received only one cocaine dose. Since it was unknown whether rats would develop a stable preference for either cocaine or sucrose over time, we tested rats for 9 days prior to any experimental interventions. We found that both male and female rats developed a rapid and sustained preference profile that is dependent upon the cocaine dose offered (Fig. S1B, D). Additionally, all groups were sensitive to the within-session progressive increase in sucrose price with generally high sucrose preference during early trials and high cocaine preference in later trials (Fig. S1. C, E).

**Figure 2.**
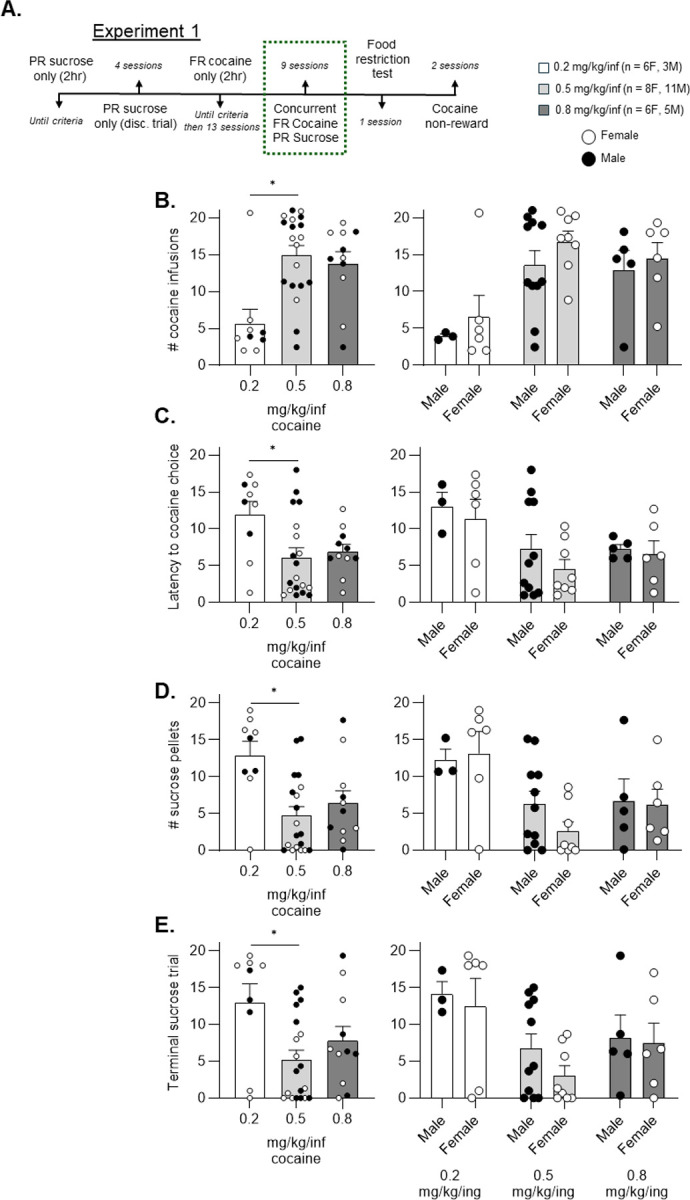
Effects of cocaine dose on cocaine versus sucrose choice in male and female rats Following single reinforcer training, male and female rats completed 9 dual reinforcer choice sessions (A). Comparisons among dose groups were made in aggregate (left) and pairwise between males and females within each dose group (right). The low cocaine dose group (0.2 mg/kg/inf) chose fewer cocaine infusions than the medium cocaine dose group (0.5 mg/kg/inf) (B). Likewise, the number of trials prior to first cocaine choice was higher in the low dose group versus the medium dose group (C). The low dose group also acquired more sucrose pellets versus the medium dose group (D). Additionally, the low dose group achieved higher effort ratios to obtain sucrose pellets as shown by a higher terminal sucrose trial compared to the medium dose group (E). *p < 0.05, Kruskal-Wallis test followed by Dunn’s multiple comparisons test.

Rats that received the medium cocaine dose (0.5 mg/kg/inf) took more cocaine infusions (Fig. 2B, Dunn’s multiple comparisons test, p = 0.006) and less sucrose pellets (Fig. 2D, Dunn’s multiple comparisons test, p = 0.003) than the low dose group (0.2 mg/kg/inf). Similarly, rats receiving the medium cocaine dose chose their first cocaine reinforcer during earlier trials (latency to cocaine choice) than the low cocaine dose group (Fig. 2C, Dunn’s multiple comparisons test, p = 0.037). As a proxy for sucrose “breakpoint” we recorded the trial in which the last sucrose pellet was earned, which represents a measure of maximal effort emitted for sucrose pellets during a session. The low-dose group achieved higher ratio requirements than the medium-dose group, as represented by higher terminal sucrose trials (Fig. 2E, Dunn’s multiple comparisons test, p = 0.024). Although there were no statistically significant differences between the high cocaine -dose group (0.8 mg/kg/inf) and the low- or medium-dose groups in any metric, overall, there was a high degree of similarity between the medium- and high-dose groups but not the low- and high-dose groups. Importantly, we did not discover any significant differences related to sex among cocaine, sucrose, latency to cocaine choice, or terminal sucrose trial (Fig. 2B, C, D, E, right side, Mann-Whitney, p > 0.05).

### Acute food restriction alters reinforcer allocation in the choice task

**Figure 3.**
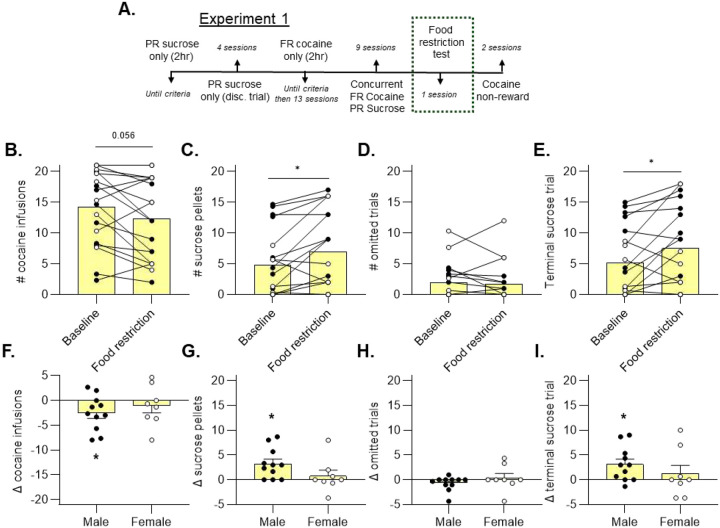
Acute food restriction shifts reinforcer allocation in male, but not female, rats Following a period of repeated choice sessions, home cage food was restricted for 24 hours prior to an additional choice session (A). Overall, there was trend toward decreased cocaine infusions after acute food restriction (B, Wilcoxon matched-pairs signed rank test, p = 0.056), but analysis of difference score for males and females separately revealed a significant decrease in males only (F, one-sample t-test, *p < 0.05). We observed a significant increase in sucrose pellets overall (C, Wilcoxon, *p < 0.05), but sex-specific analysis revealed a significant increase in the male group only (G, one-sample t-test, *p < 0.05). The number of omitted trials did not change after acute food restriction (D, H). The terminal sucrose trial increased overall (E, Wilcoxon, *p < 0.05), but sex-specific analysis revealed a significant difference in males only (I, one-sample t-test, *p < 0.05).

Next, we sought to understand if choice allocation could be shifted through manipulating hunger state. Forrats from the medium cocaine group (0.5 mg/kg/inf), food was removed from each home cage such that each animal was without standard chow for 24 hours following the 9^th^ choice session (Fig. 3A). We hypothesized that acute food restriction would inflate the value of sucrose, leading to increased sucrose choice and decreased cocaine choice. We compared the averaged data from the last 3 choice sessions (baseline) to the choice session immediately following 24 hours of food restriction.

The overall data, including both male and female rats, did not demonstrate a statistically significant effect on cocaine choice, despite a trend toward a reduction (Fig. 3B, Wilcoxon test, p = 0.056). However, sucrose choice was significantly increased (Fig. 3C, Wilcoxon test, p = 0.010). After splitting data by sex, we computed a difference score for each metric (food restricted value minus baseline value). The difference score satisfied normality assumptions; therefore, parametric statistical testing was used for comparisons. We found that males (p = 0.038), but not females (p = 0.477), deceased their cocaine choice following acute food restriction (Fig. 3F, one-sample t-test). There was no significant difference when directly comparing the cocaine choice difference scores between male and female rats (Fig. 3F, unpaired t-test, p > 0.05).

Sucrose choice was increased overall (Fig. 3C, Wilcoxon test, p = 0.010), indicating that motivation for sucrose increased after acute food restriction. However, independent analysis of each sex revealed that only male rats significantly increased sucrose choice under these conditions (Fig. 3G, one-sample t-test, p = 0.006). When comparing sucrose choice difference scores between male and female rats, we did not find a significant difference (Fig. 3G, unpaired t-test, p > 0.05). Interestingly, female rats tend to obtain more sucrose pellets and reach higher ratio requirements when sucrose is offered as the sole reinforcer (Fig. S2). This finding makes it unlikely that females are less motivated to obtain sucrose than males.

We did not observe an overall increase in omissions after acute food restriction (Fig. 3D, Wilcoxon test, p = 0.510). Moreover, there were no sex differences in omitted trials (Fig. 3H, unpaired t-test, p > 0.05)

To better quantify motivation for sucrose, we recorded the terminal sucrose trial for each rat, which represented the highest PR value achieved for sucrose in each session. Overall, we observed that the last sucrose choice occurred at later trials after acute food restriction (Fig. 3E, Wilcoxon test, p = 0.031). Difference scores for terminal sucrose trial analyzed separately for each sex revealed that males (p = 0.013), but not females (p = 0.504), showed a higher terminal sucrose trial after food restriction (Fig. 3I, one-sample t-test). The change in the terminal sucrose trial value relative to baseline did not significantly differ between male and female rats (Fig. 3I, unpaired t-test, p > 0.05).

In an attempt to understand if choice behavior could generalize across different strain types, we trained a small exploratory cohort (n=4) of female Long Evans rats following the same protocol as detailed in Experiment 1. Interestingly, we found that the Long Evans rats took much less cocaine and more sucrose than the SD females tested at the same cocaine dose (0.5 mg/kg/inf). Therefore, instead of testing acute food restriction, we decided to offer an abundance of sucrose in the operant chamber (non-contingent access) for 10 minutes immediately prior to a choice session termed the “satiety session”. We found a trend towards increased cocaine choice (Fig. S3A, paired t-test, p = 0.064), a significant decrease in sucrose choice (Fig. S3B, paired t-test, p = 0.037), and no change in omitted trials (Fig. S3C, paired t-test, p = 0.257).

### Cocaine non-reward shifts allocation towards sucrose and away from cocaine choice

Following the food restriction test, animals were returned to an *ad libitum* diet, an additional choice session was conducted, and a subset of rats proceeded to the next phase of testing. This phase incorporated a new response contingency termed cocaine non-reward. Under this contingency, participation in an identical choice session, but when the cocaine reinforcer was chosen, no cocaine was delivered despite termination of the trial. Thus, the choice was between sucrose pellets and cocaine-associated cues.

**Figure 4.**
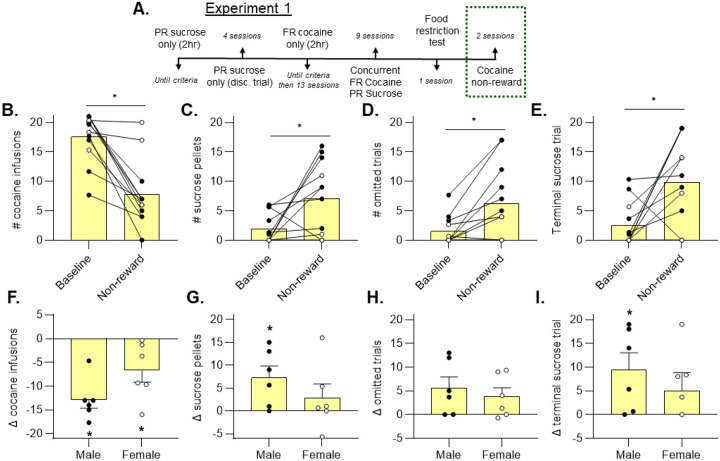
Cocaine non-reward conditions lead to decreased cocaine choice in male and female rats, yet only males reallocate towards sucrose Choice behavior was examined when the cocaine reinforcer was withheld upon cocaine choice creating conditions of unreinforced cocaine choice (non-reward) (A). Following a single learning session under these conditions, rats significantly decreased the number of cocaine infusions chosen (B, Wilcoxon, *p < 0.05), an effect that was present in both sexes (F, one-sample t-test, *p < 0.05). The number of sucrose pellets was increased overall (C, Wilcoxon, *p < 0.05), but this effect was limited to the male group (G, one-sample t-test, *p < 0.05). There were more omissions in the cocaine non-reward conditions (D, Wilcoxon, *p < 0.05), but analysis of each sex individually did not reveal a significant effect (H, one-sample t test, p > 0.05). Sucrose was chosen later in the session (E, Wilcoxon, *p < 0.05), but this effect was shown to be significant in the male group only (I, one-sample t-test, *p < 0.05).

Two sessions were conducted using the cocaine non-reward contingency (Fig. 4A). The first non-reward session served as a learning opportunity for the rats to become familiar with the new contingency and was not included in analyses. Data from the 2^nd^ cocaine non-reward choice session were compared to baseline values to determine the effects of cocaine non-reward on choice behavior. We hypothesized that the withholding of the cocaine infusion would significantly devalue cocaine choice and lead to an increase in sucrose choice when compared to baseline preference.

Overall, we found that cocaine choice was significantly reduced by cocaine non-reward conditions (Fig. 4B, Wilcoxon test, p = 0.001). Further analysis by sex indicated that both male (p = 0.001) and female (p = 0.041) rats reduced their cocaine choice under non-reward conditions (Fig. 4F, one-sample t-test). There were no differences between sexes when measuring the difference of cocaine choice before and after initiating the cocaine non-reward contingency (Fig. 4F, unpaired t-test, p > 0.05).

Overall, sucrose choice was increased following implementation of the cocaine non-reward contingency. However, we found that males (p = 0.034), but not females (p = 0.378), increase sucrose choice when tested under non-reward conditions (Fig. 4G, one-sample t-test). Omissions were increased overall (Fig. 4D, Wilcoxon test, p = 0.008), but when analyzing each sex separately, neither group displayed significantly more omissions versus baseline.

As another measure of motivation for sucrose pellets, we assessed terminal sucrose trial values. Overall, the terminal sucrose trial value increased under cocaine non-reward conditions (Fig. 4E, Wilcoxon test, p = 0.027). Similar to sucrose choice, we found that the terminal sucrose trial number only significantly increased in males (p = 0.042), and not females (p = 0.239), suggesting a lack of reallocation by females in response to the cocaine non-reward contingency (Fig. 4I, one-sample t test).

### Cocaine choice punished by aversive white noise (AWN) elicits sex-specific behavioral allocation

**Figure 5.**
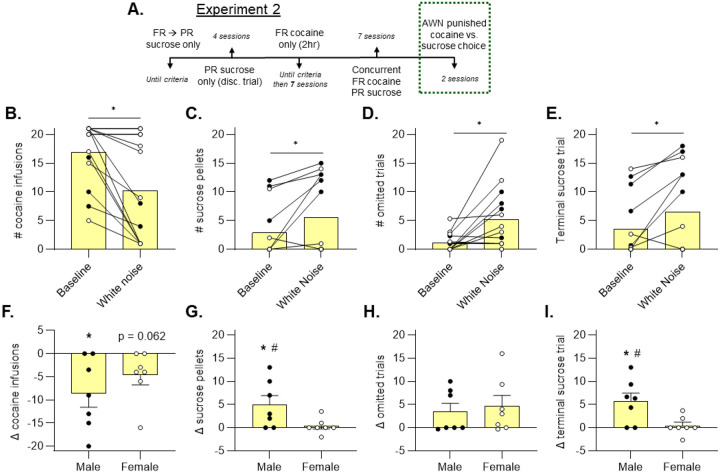
Aversive white noise-punished cocaine choice results in sex-specific reallocation strategies Experiment 2 involved measuring choice behavior when cocaine choice was punished by an aversive white noise (A). There was an overall decrease in cocaine infusions in response to the punishment contingency (B, Wilcoxon, *p < 0.05). This effect was significant in males (F, one-sample t-test, *p < 0.05, and a strong trend was found for females (p = 0.062). There was an overall increase in sucrose pellets earned (C, Wilcoxon, *p < 0.05). Males showed a significant increase from baseline (G, one-sample t-test, *p < 0.05) but also showed a greater increase in sucrose pellets when compared to females (G, unpaired t-test, #p < 0.05). There was a general increase in omissions (D, Wilcoxon, *p < 0.05), but there was no individual effect found within each sex. Similarly to the number of sucrose pellets, there was an overall increase in the terminal (latest) trial during which sucrose was earned (E, Wilcoxon, *p < 0.05). This effect was restricted to males (I, one-sample t-test, *p < 0.05), and males showed a greater change in terminal sucrose trial when compared to females (I, unpaired t-test, #p < 0.05).

Since we found that rats updated their reinforcer preferences under conditions of acute food restriction and cocaine non-reward, we next tested whether rats respond similarly when cocaine was delivered followed by punishment (i.e., aversive white noise; AWN). A separate cohort of male (n=7) and female (n=7) rats were used for this study. All rats received cocaine at the 0.5 mg/kg/inf dose. After single reinforcer training and 7 concurrent choice sessions, the response contingency changed such that each cocaine infusion was now punished by a 10 second burst of 90dB white noise (Fig. 5A). An initial learning session was performed after the 7-session concurrent choice period in the same format as the typical choice sessions to teach the rats that cocaine infusions would now be paired with an aversive white noise. This learning session was not included in the analysis. Next, an additional test session was performed after rats had an opportunity to update their expectations to the new punishment contingency.

We observed that AWN punishment led to a decrease in cocaine choice (Fig. 5B, Wilcoxon test, p = 0.002) compared to baseline values (avg. last 3 choice sessions). Further analysis of each sex independently revealed that males (p = 0.026), but not females (p = 0.062), reduced their cocaine choice after punishment (Fig. 5F, one sample t-test). Next, we examined reallocation towards sucrose during punished cocaine choice. We found an overall increase in sucrose choice when cocaine choice was punished by AWN (Fig. 5C, Wilcoxon test, p = 0.023). However, we observed a marked divergence between male and female rats. Male rats increased their sucrose choice significantly more than female rats after initiation of the punished cocaine contingency (Fig. 5G, unpaired t-test, p = 0.041). Moreover, male (p = 0.041), but not female (p = 0.588), rats exhibited a significant increase in sucrose choice (Fig. 5G, one sample t-test). These data suggest that males display greater reallocation towards sucrose when cocaine choice is punished by AWN.

We found an overall increase in omitted trials after cocaine punishment (Fig. 5D, Wilcoxon test, p = 0.010). When analyzed independently, neither sex showed a significant difference in omissions from baseline (Fig. 5H, one sample t-test, p > 0.05).

Similar to sucrose choice, we observed an overall increase in the terminal sucrose trial value (Fig. 5E, Wilcoxon test, p = 0.023), and there was a significant difference between male and female rats (Fig. 5I, unpaired t test, p = 0.020). The terminal sucrose trial was increased by AWN punishment in male rats (p = 0.020) but not female rats (p = 0.586; Fig. 5I, one sample t-test). These data suggest that not only do males take more sucrose after cocaine is punished, but they exert more effort to obtain sucrose as well.

## Discussion:

The present study explores a novel cocaine versus sucrose choice task that promotes within-session mixed choice behavior. By leveraging an asymmetric reinforcer schedule, exclusive sucrose choice could be mitigated. This approach is relatively simple and can be easily applied to assess the impact of environmental or pharmacological manipulations. We found that male and female rats allocate responses towards cocaine or sucrose similarly, but when reinforcer contingencies are altered, males tend to reallocate, while females either do not change behavior or decide to omit trials. First, we showed that male rats increase their sucrose choice under conditions of acute food restriction, while female rats do not. Second, we show that although both male and female rats decrease cocaine choice in response to a cocaine non-reward contingency, only male rats increase sucrose choice. Lastly, we demonstrate that under conditions of punished cocaine choice, it is again male, but not female, rats that reallocate behavior away from cocaine and towards sucrose. Together, we show that diverse contingency manipulations elicit sex-dependent alterations in cocaine versus sucrose choice which are not present under baseline conditions.

### Interpretation among previous cocaine versus alternative reinforcer models

Many reports have characterized rodent responses in choice situations between cocaine and non-drug alternatives. On one hand, evidence strongly supports the notion that sucrose and other non-drug reinforcers are strongly preferred by most rats compared to drugs that are commonly misused by humans (Lenoir, Serre, Cantin, and Ahmed 2007; Cantin et al. 2010). These findings have elicited skepticism about whether rodents can serve as viable preclinical models since they readily give up drug access in favor of non-drug reinforcers. However, others have demonstrated that non-drug preference may be overcome by manipulating the magnitude of either reinforcer dose or price (for review see (Kearns 2025) and also (Marcus and Banks 2025)). Additional studies have pointed towards a potential temporal mismatch between drug and non-drug reinforcers with regards to dopamine signaling in reward centers (Canchy et al. 2021). Moreover, modifying the interval between the choice action and non-drug reinforcer delivery can skew preference towards drug (Canchy et al. 2021; Venniro, Panlilio, Epstein, and Shaham 2021).

We posit that both schools of thought are likely valid, with rats possessing a strong drive for non-drug rewards that can be offset by altering the nature of choice contingency. To leverage this understanding, we used an asymmetric reinforcement schedule that biases towards initial non-drug choice, while creating gradual incentive for drug choice as the sucrose option becomes progressively more expensive. There is only one existing study to date that has investigated within-session asymmetric reinforcer schedules involving cocaine choice in rats (Cantin et al. 2010) and none, to our knowledge, that have leveraged this format to study punished drug choice.

To date, one of the most compelling cocaine versus non-drug reinforcer models that has been applied to human subjects involves offering the choice between cocaine or a monetary incentive (Lile et al. 2020; Lile et al. 2016; Stoops et al. 2012; Stoops, Lile, and Rush 2010). As the effort requirement for a monetary reward increases across sessions while keeping the cocaine requirement at a low, fixed ratio, people tend to choose more cocaine (Stoops et al. 2012). Interestingly, when both cocaine and money are offered on a progressive ratio within a given session, drug preference is highly dependent upon the cocaine dose, with high money choice observed at low cocaine doses, mixed choice at moderate cocaine doses, and high cocaine choice at high cocaine doses (Lile et al. 2016). We observed a similar effect in rats during our within-session effort manipulation, suggesting that rodent behavior in our paradigm may be a suitable approximation for human tendencies.

We found that cocaine choice is relatively low when the sucrose requirement is low, while cocaine choice is high when sucrose requirement is high. Cantin et al. describe a similar pattern of behavioral allocation when measuring within-session preference for sweet water versus intravenous cocaine whereby preference for sweetened water was reversed by selectively increasing the effort requirement for sweet water throughout the session (Cantin et al. 2010). The authors demonstrated that rats switch their preference away from sweet water and towards cocaine depending upon the saccharin concentration of the water. Although we did not manipulate the magnitude of the sucrose reinforcer, our results, together with this previous work, suggest that within-session preference between palatable reinforcement and cocaine can be manipulated by the changing the intensity of either reinforcer. Similarly, Venniro and colleagues describe a between-session manipulation of both price and delay of the non-drug reinforcer (social interaction), displaying significant effects of non-drug price and reinforcer delay (Venniro, Panlilio, Epstein, and Shaham 2021).

Our results demonstrate that cocaine choice at each sucrose price is dependent upon the cocaine dose offered. In a study by Thomsen et. al., the authors describe a cocaine dose relationship between cocaine choice and a palatable food reinforcer (Ensure) (Thomsen, Barrett, Negus, and Caine 2013). A within-session protocol was used that involved manipulating the dose of cocaine available to assay choice preference. As the dose of cocaine increased during the session, so did cocaine preference, yet once the cocaine dose reached its peak (1 mg/kg/inf), the number of completed trials dropped precipitously. In the present study, we manipulated cocaine dose as a between-subjects factor, such that each rat only experienced a single cocaine dose. Nevertheless, we observed cocaine preference measures that align with published reports of within-session cocaine dose changes, although we found no significant differences between a moderate (0.5 mg/kg/inf) and a relatively high (0.8 mg/kg/inf) dose. It remains possible that a higher dose (1+ mg/kg/inf) may elicit greater cocaine choice, however, it is also likely that a high dose would increase omissions given the discrete trial nature of the task.

### Acute food restriction increases while sucrose pellet satiety decreases sucrose choice

A bout of acute food restriction is likely to increase the desire for calories and therefore increase motivation for sucrose pellets. Our data corroborate this idea, showing that one day of food restriction is sufficient to increase sucrose choice in male rats. These findings are consistent with a report from Cantin et al. who found that chronic food restriction promoted sucrose vs. cocaine choice (Cantin et al. 2010). Notably, in that study, the investigators reported that while chronic food restriction increased sucrose choice, it had a greater effect compared to rats choosing between cocaine and saccharin. Sucrose contains calories, in contrast to saccharin, which is a sweet, but non-caloric reinforcer. These findings indicate that physiological needs may influence drug choice in rats. By contrast, work by Negus in non-human primates found that restricting male rhesus monkeys to half of their normal diet did not alter cocaine choice (Negus 2003). However, the food available during the cocaine versus food choice task was a standard food pellet rather than a sweetened reward, which might be important for the emergence of the effect.

While food restriction failed to influence cocaine choice, the study by Negus found that pre-feeding increased cocaine choice early in the cocaine dose response curve, suggesting that satiety may shift preference away from food to drug (Negus 2003). In a preliminary experiment, we reproduced these findings in our choice protocol. When we tested a small group of female Long Evans rats for choice after they were provided ten minutes of unlimited access to sucrose pellets, we observed a shift away from sucrose to cocaine choice. Overall, our data suggest that acute food restriction promotes sucrose choice, while food satiety shifts behavior towards cocaine choice.

While the translational relevance of these studies is unclear, it is tempting to draw parallels between preclinical findings demonstrating reductions in cocaine choice during food restriction and increased cocaine choice under conditions of satiety and the influence of available financial resources to drug consumption in humans. Such comparisons, while limited, may have implications for understanding the relationship between economic status and drug use, as well as contingency management-based interventions.

### Male, but not female rats, reallocate behavior towards sucrose when cocaine choice is punished

Despite questions about validity (George, Ahmed, and Gilpin 2022), punished drug self-administration has been used as a measure of compulsive drug taking in preclinical studies. Contingent presentation of footshock or delivery of aversive agents (e.g., histamine, kappa opioid agonists, etc.,) has been used as punishment for drug taking in choice models (Pelloux, Murray, and Everitt 2015; Minervini, Tye, Ghodrati, and France 2021). These interventions have been reported to abolish drug taking behavior in most rats (depending on intensity/dose of the aversive agent), while a small minority of rats retain some drug taking behavior. The availability of sucrose as an alternative reinforcer has been shown to facilitate the punishment of cocaine reinforcement by aversive footshock (Pelloux, Murray, and Everitt 2015). Interestingly, this finding was reported in male rats only. In our study, we find that male rats reallocate behavior away from cocaine and toward sucrose and, to a lesser extent, omissions, while females reallocate towards omissions only. While our data from male rats align with previous work, our observations in female rats challenge existing views.

It has recently been reported that negative reinforcement can reduce drug taking in under choice conditions. Marcus et al. demonstrated that when provided a choice between cocaine and negative reinforcement (shock avoidance or escape), rats strongly prefer the negative reinforcer (or omissions) over cocaine across multiple effort levels (Marcus and Banks 2023). These results showcase the powerful incentive to avoid aversive stimuli in the context of drug choice. Our model of punished drug choice exhibits qualities of positive and negative reinforcement such that sucrose choice results in acquisition of a palatable reinforcer as well as avoidance of the aversive white noise presentation. The dual reinforcement aspect of our model could explain the overall tendency for rats to reallocate rather than to omit trials in our study.

### Study limitations

To properly interpret our study, there are several factors that should be considered. First, we did not dedicate a group of rats to receive a saline dose in Experiment 1. When rats reach higher sucrose effort levels, they tend to switch to cocaine. To better understand if this switch represented motivation for cocaine rather than an act of frustration, a comparison of the behavior of the low-dose group to a group choosing between sucrose and saline would be useful. We predict that the saline group would acquire a sucrose pellet during almost every trial, similar to how rats behave during the sucrose-only single reinforcer training. A similar critique could be made in that we did not test a high enough dose of cocaine. For example, in Experiment 1 data from the medium and high dose groups were very similar. This could be due to similar reinforcing properties of the two doses, or it could represent a limit to the reinforcing value of cocaine in this discrete trial format. Examining choice behavior when cocaine is offered at a dose of 1 mg/kg/infusion or higher may provide clarity on the relationship between cocaine dose and choice preference in this model. Another consideration is that we did not examine cocaine-only responding in the discrete trial format. This information would be useful to understand how many omissions could be expected when an alternative reinforcer is not present. Future studies will involve testing under discrete trial conditions for both reinforcers individually.

## Conclusion

In conclusion, here we introduce and characterize a novel cocaine vs. sucrose choice protocol that can be deployed to assess how various factors influence reinforcer preference. We demonstrate that choice behavior in this protocol changes, as predicted, in response to variation in cocaine dose, drug removal, food restriction/satiety, and punishment. Moreover, testing using this approach reveals that male and female rats exhibit unique reallocation strategies in response to changing contingencies. Future studies will extend our findings to other misused drugs and apply this approach to guide medication development, understand neurobiological mechanisms that govern drug choice, and further explore the influence of factors that contribute to SUDs.

## Supplementary Material

1

Supplementary Files

This is a list of supplementary les associated with this preprint. Click to download.

- Nowaketal2025Supplementary.docx
