# Supplementary material for: Assessing behavioral reallocation after acute environmental manipulations using an asymmetric cocaine versus sucrose choice task in male and female rats
Source: Res Sq. 2025 Oct 30:rs.3.rs-7802392. Preprint. [Version 1] doi: 10.21203/rs.3.rs-7802392/v1 (PMC12636728; doi:10.21203/rs.3.rs-7802392/v1)
Supplement: 1 [file NIHPPRS7802392V1-supplement-1.pdf]

SUPPLEMENTARY MATERIALS

Figure S1. Between and within-session response data

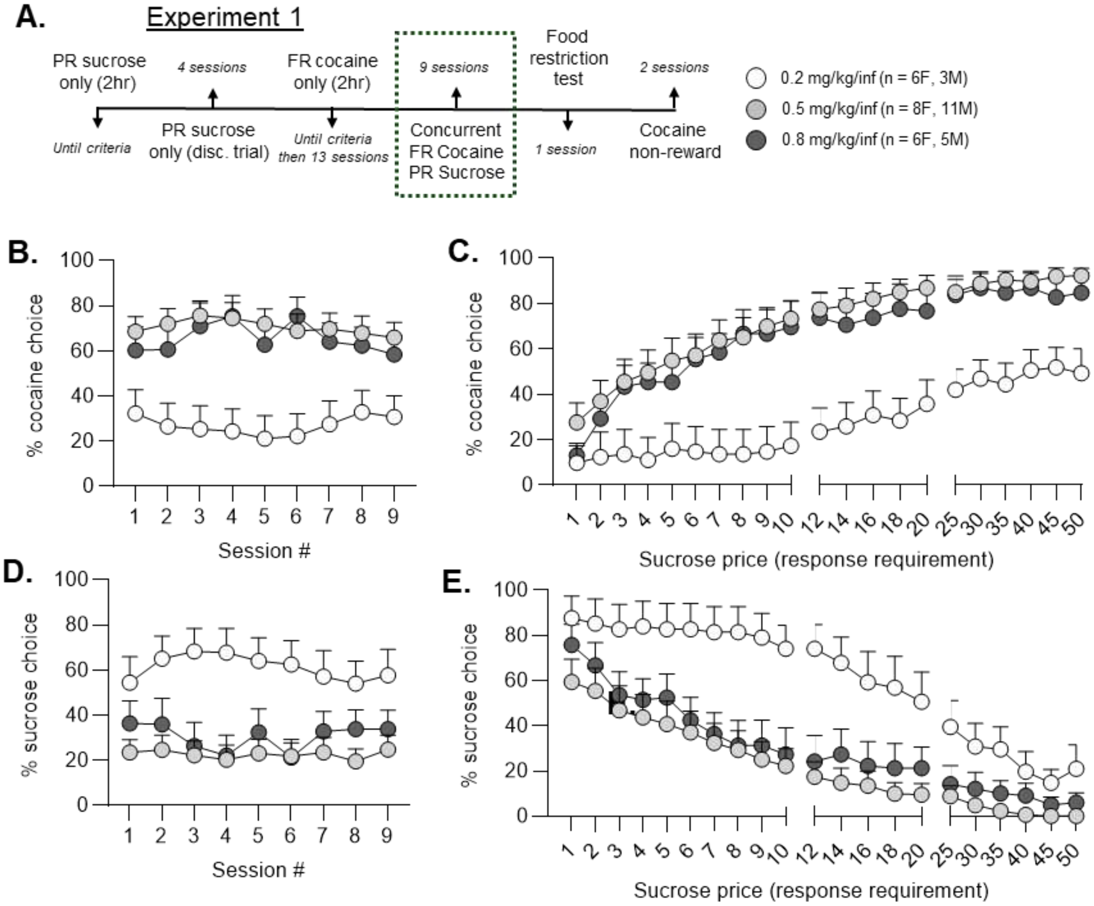

After single-reinforcer training, rats completed 9 daily choice sessions (A). Cocaine choice was stable over the testing period for all groups (Fig. S1B, Friedman test, $p > 0.05$). Within each session, there was a significant effect of sucrose price on cocaine choice when data was averaged by trial for all groups (Fig. S1C, Friedman test, $p < 0.05$). We determined that there was no effect of session on sucrose choice (Fig. S1D, Friedman test, $p > 0.05$), except in the 0.8 mg/kg/inf cocaine dose group (Friedman test, $p = 0.045$). However, further testing using Dunn's multiple comparisons test revealed no significant differences between sessions (Fig. S1D, Dunn's multiple comparisons test, $p > 0.05$). Similar to cocaine choice, we discovered a significant effect of sucrose price on sucrose choice across trials (Fig. S1E, Friedman test, $p < 0.05$). Symbols denote cocaine dose group average plus standard error.

Figure S2. Sucrose-only progressive ratio training

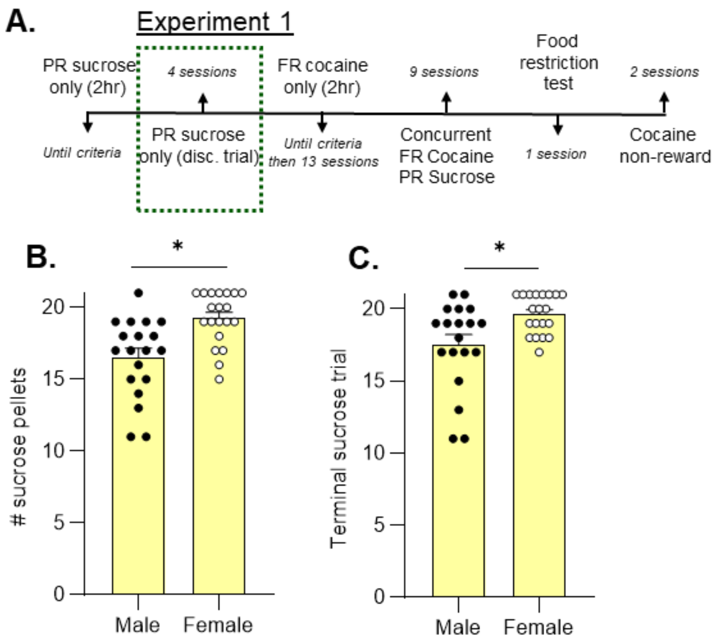

In Experiment 1, rats were first trained to respond under a progressive ratio schedule of sucrose pellet reinforcement with no time restriction for each ratio requirement (2-hour limit for each session). After responding was acquired, rats completed 4 discrete trial sucrose-only training sessions that were structured identically to the dual-reinforcer choice sessions, except that only the sucrose lever was inserted (Fig. S2A). Data from the last discrete trial session are shown with bars indicating the mean plus standard error and each symbol represents one animal (Fig. S2B, C). We observed that female rats achieved more sucrose pellets than males (Fig. S2B, Mann-Whitney test, *$p < 0.05$). Additionally, female rats are willing to exert more effort for their last sucrose pellet (terminal sucrose trial) than male rats (Fig. S2C, Mann-Whitney test, *$p < 0.05$).

Figure S3. Effects of acute sucrose satiety on choice behavior

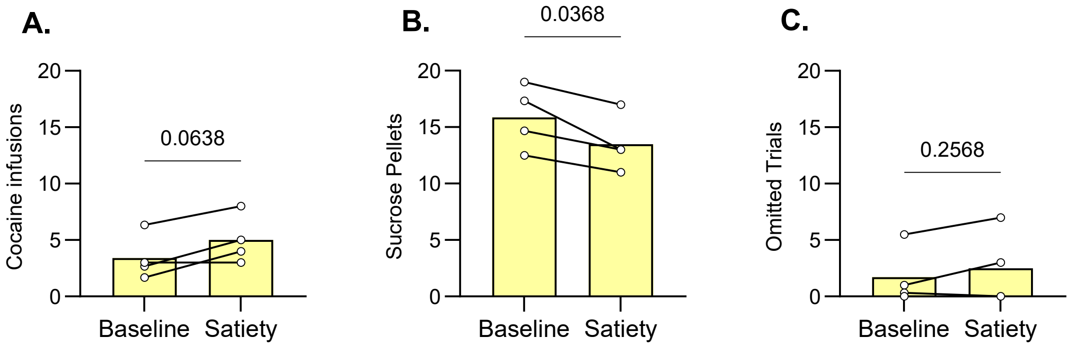

In a small cohort of female Long-Evans rats (n=4), we examined the effect of acute sucrose satiety on choice between sucrose and cocaine (0.5 mg/kg/inf). Data shown is the mean baseline (avg. last 3 choice sessions) versus single satiety session data. Each pair of symbols and connecting line represents 1 animal. We found a trend towards increased cocaine choice (Fig. S3A, paired t-test, $p = 0.064$), a significant decrease in sucrose choice (Fig. S3B, paired t-test, $p = 0.037$), and no change in omitted trials (Fig. S3C, paired t-test, $p = 0.257$).
